# Pressure-Induced Crystallization and Phase Transformation of *Para*-xylene

**DOI:** 10.1038/s41598-017-05639-9

**Published:** 2017-07-13

**Authors:** Yanzhi Bai, Zhenhai Yu, Ran Liu, Nana Li, Shuai Yan, Ke Yang, Bingbing Liu, Dongqing Wei, Lin Wang

**Affiliations:** 1 Department of Physics and Astronomy and College of Life Science and Biotechnology, Shanghai Jiaotong University, Shanghai, 200240 People’s Republic of China; 2 Center for High Pressure Science and Technology Advanced Research, Shanghai, 201203 People’s Republic of China; 3 State Key Laboratory of Superhard Materials, Jilin University, Changchun, 130012 People’s Republic of China; 4 Shanghai Institute of Applied Physics, Chinese Academy of Sciences, Shanghai, 201203 People’s Republic of China; 5 High Pressure Synergetic Consortium, Geophysical Laboratory, Carnegie Institution of Washington, Argonne, Illinois 60439 United States of America

## Abstract

Static pressure is an alternative method to chemical pressure for tuning the crystal structure, bonds, and physical properties of materials, and is a significant technique for the synthesis of novel materials and fundamental research. In this letter, we report the crystallization and phase transformation of *p*-xylene under high pressure. Our optical micrographic observations and the appearance of lattice modes in the Raman and infrared (IR) spectra indicated that *p*-xylene crystallizes at ∼0.1 GPa. The X-ray diffraction (XRD) pattern at 0.84 GPa suggests that the crystallized *p*-xylene had a monoclinic phase with the Cc(9) space group. The sharp shrinkage of the lattice at ~13 GPa and the solid state of the decompressed sample we observed suggests a new crystalline phase of *p*-xylene. The *in situ* XRD showed that the new crystalline phase was still a monoclinic structure but with a different space group of C2(5), indicating that a phase transition occurred during further compression. The mass spectrometry experiment confirmed phase transition polymerization, with mainly trimer and tetramer polymers. Our findings suggest an easy and efficient method for crystallizing and polymerizing *p*-xylene under high pressure.

## Introduction

High-pressure physics and chemistry offer innovative strategies to synthesize new materials for various applications^1–3^. One such strategy is the crystallization of liquid as an alternative to crystallization at low temperature. The pressure-induced synthesis of crystals is industrially important because eliminating catalysts produces more pure products. Thus, pressure is a powerful tool to produce crystalline organic compounds. Many examples of this have been investigated by several groups^4–6^. Piermarini *et al*. found that carbon tetrachloride solidifies into solid Ib at 0.13 GPa, transforms into another solid phase II at 0.4 GPa, and finally Phase III at 0.7 GPa^7^. Intensive research was conducted on the carbon tetrachloride using XRD under high pressure to define its detailed structures.

*Para*-xylene (*p*-xylene) is an aromatic hydrocarbon consisting of a benzene ring with two methyl substituents at opposite positions. The symmetry is D_2h_ (this designation ignores the methyl group hydrogen atoms). Polymerized *p*-xylene is an industrially important polymer, which is primarily used as a basic raw material in the manufacturing of terephthalic acid (TPA), purified terephthalic acid (PTA), and dimethyl-terephthalate (DMT). These, in turn, are used to manufacture polyethylene terephthalate (PET)-saturated polyester polymers. These polyesters are lightweight, shatter-resistant, and have high tensile strength. With the increasing global demand for TPA, the need for polymerized *p*-xylene production is also growing rapidly. The high yield and high stability of the polymer make this process appealing for large-scale applications. However, the crystallization, polymerization, and characterization of *p*-xylene under high pressure have not yet been reported.

Here, we report our crystal growth observations and pressure-induced phase transformation of *p*-xylene up to ~31 GPa in a diamond anvil cell (DAC) at room temperature (24–28 °C in our lab). As the pressure was carefully manipulated, *p*-xylene crystallized at ~0.1 GPa. We also investigated the crystallized *p*-xylene using XRD. Our preliminary Raman and IR analysis indicated that the pressure-induced phase transformation (polymerization) occurred around 13 GPa. Remarkably, the polymerized solid phase was quenchable to ambient conditions and confirmed by mass spectrometry experiments on the decompressed samples.

## Results and Discussions

**Figure 1:**
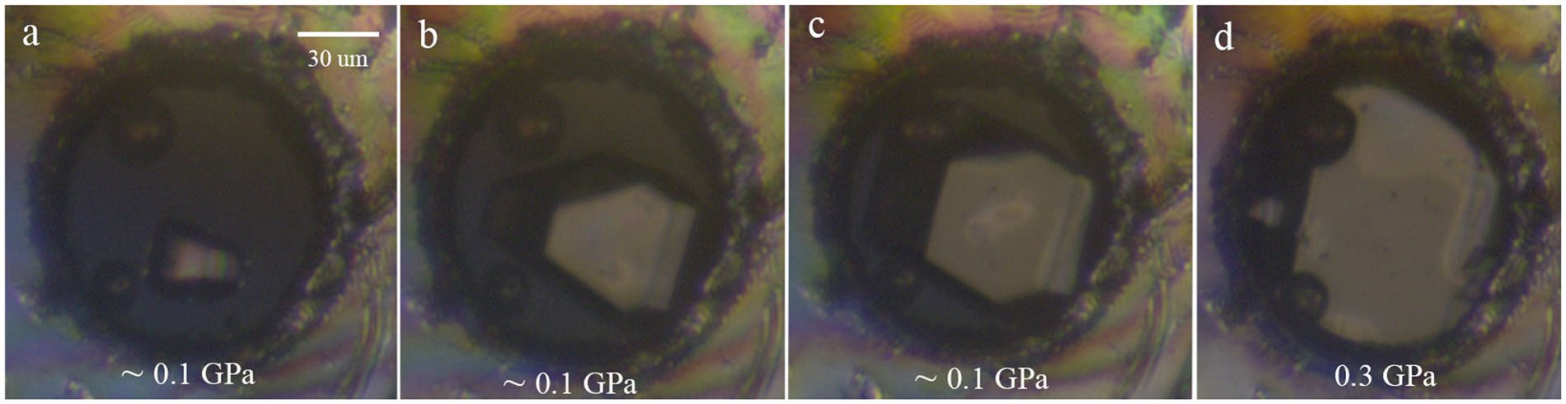
Images of the equilibrium forms of the *p*-xylene grown in the DAC. Temperature: 24–28 °C. The two spheres on the left part of the hole are the ruby balls for *in situ* pressure measurements.

Liquids usually crystallize at a defined pressure. Many organic solvents have been crystallized via pressure, including odd-numbered normal alkanes (carbon number: 9, 11 and 13)^8^, cyclohexane^9^ and carbon tetrachloride^10^. However, the properties of crystallized *p*-xylene remain unknown. Here, we studied the pressure-induced crystallization of *p*-xylene with optical microscopy and XRD at room temperature. The *p*-xylene was compressed above the freezing pressure of the liquid. Then, we obtained a single crystal through controlling the pressure by repeating the decompression and compression cycles. The freezing pressure of *p*-xylene was ~0.1 GPa. Images of the equilibrium forms of the *p*-xylene grown in the DAC are shown in Fig. 1(a–c). Finally, the crystals fully accommodated the DAC at a higher pressure (0.3 GPa), as shown in Fig. 1(d).

In order to better characterize the crystallized *p*-xylene, XRD patterns were performed under high pressure and room temperature. The first trace was measured at 0.84 GPa. Independent Raman measurements of *p*-xylene from ~0.1 to ~1.1 GPa were performed, and no detectable variation was observed between 0.1 and 0.84 GPa. Therefore, the sample at 0.84 GPa was the same as the crystal formed at ~0.1 GPa.

**Figure 2:**
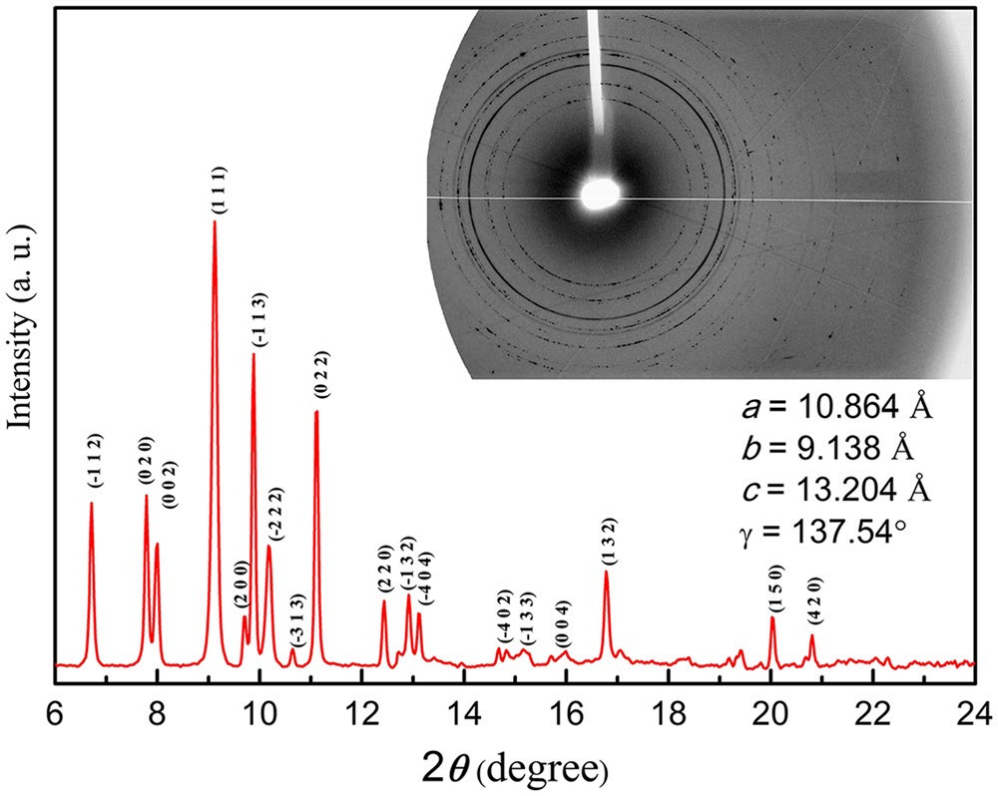
Powder X-ray diffraction pattern of *p*-xylene at 0.84 GPa. The calculated diffraction lines for this crystal structure are assigned. The inset shows the 2D diffraction image.

The XRD patterns and typical two-dimensional (2D) diffraction image of the *p*-xylene obtained at 0.84 GPa are shown in Fig. 2. The appearance of diffraction rings in the resulting 2D diffraction image suggested a crystal phase and confirmed that the *p*-xylene crystallized completely at this pressure. The diffraction peaks were analyzed and the *hkl* reflection lines are highlighted in Fig. 2. The results indicated that the crystallized *p*-xylene had a monoclinic symmetry with cell dimensions of *a* = 10.864 Å, *b* = 9.138 Å, *c* = 13.204 Å, and γ = 137.54°. This phase differed from that determined at low-temperature in another study^11^; possibly due to the different pressure and temperature conditions of the two studies or the perdeuteration (D) influence.

**Figure 3:**
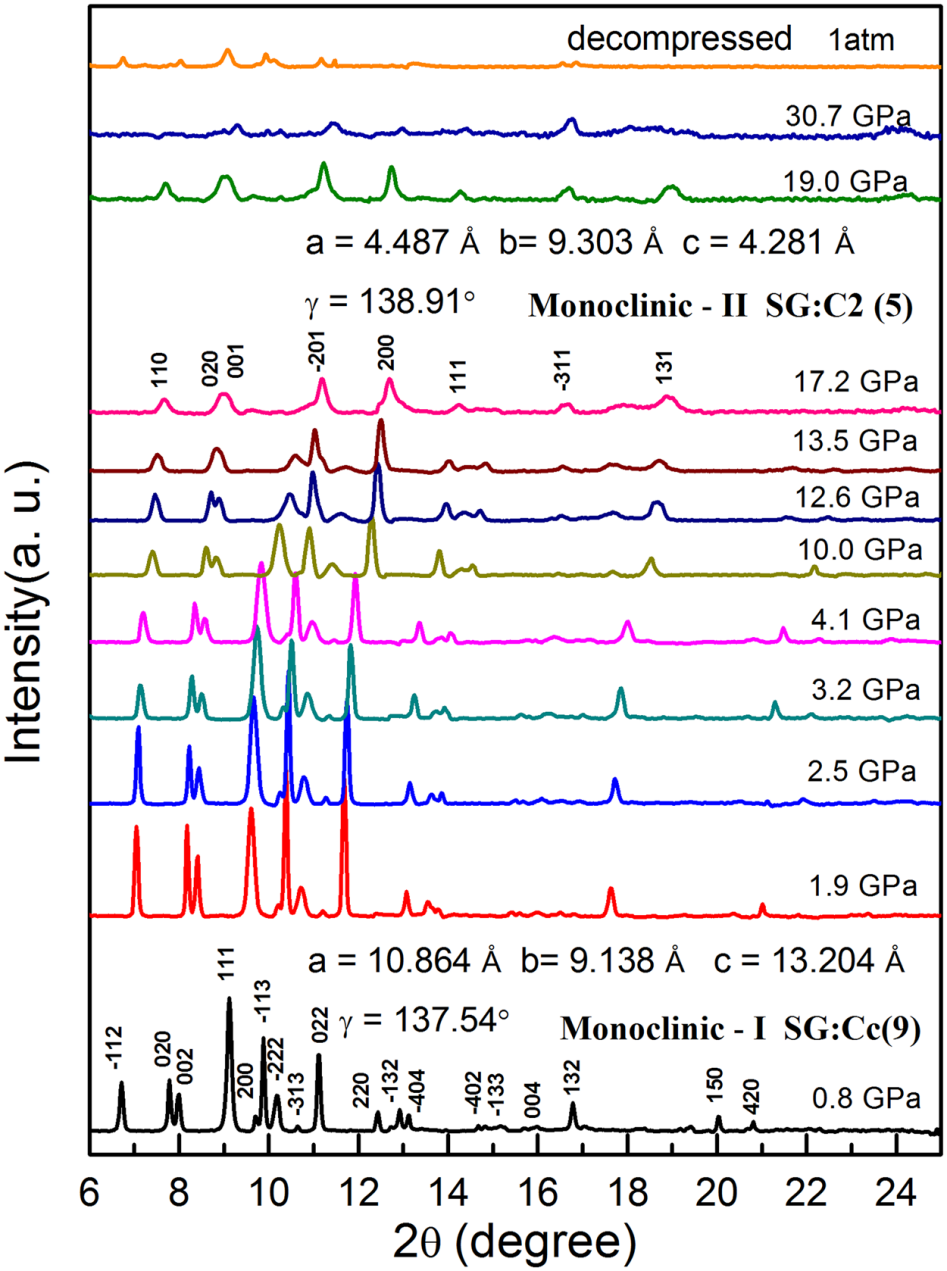
Pressure evolutions of the X-ray diffraction patterns of *p*-xylene up to 30.7 GPa. The Bragg peaks’ indexes and the cell parameters at 0.8 and 17.2 GPa are shown in the figure, respectively.

XRD experiments of *p*-xylene were performed up to 30.7 GPa at room temperature (Fig. 3). All of the diffraction peaks broadened as the pressure increased, possibly because of the non-hydrostatic pressure in the sample chamber. The Bragg peaks shifted towards high angles (low *d*-spacing) due to lattice compression. During compression up to 10.0 GPa, no obvious change besides the peak broadening was observed in all diffraction peaks, indicating that the sample remained in a low-pressure monoclinic phase. It is worth noting that there were clear changes in the intensity of the low-angle Bragg peaks (compare −112 with 020 and 002, and 111 with −113). The diffraction peaks’ relative intensities change was most probably due to the preferred orientation of the crystals at high pressure.

As the pressure reached 13.5 GPa, the intensities of (111) and (−222) started to decrease and eventually disappeared at 17.2 GPa, suggesting the sample completely transformed into a new phase. In the range of 13.5–17.2 GPa, two phases coexisted. Our analysis of the XRD results proposes that the sample was still in a monoclinic structure but with a different space group of C_2_(5) at 17.2 GPa, and the cell dimensions were *a* = 4.487 Å, *b* = 9.303 Å, *c* = 4.281 Å, and γ = 138.91°. All the diffraction peaks of this new monoclinic phase (at 17.2 GPa) are also indexed in Fig. 3. The basic regular molecular arrangement in *p*-xylene forms a lattice, with the *p*-xylene molecules occupying the lattice site. The parallel arrangement of the hexagonal faces of the benzene rings with a slight shift should be just one of the arrangement forms in *p*-xylene. However, the limited information obtained from powder XRD did not allow us to determine a reliable crystal structure (symmetry, atomic position etc.). As the pressure increased above 19 GPa, the intensity of the XRD pattern became weaker and weaker, because the sample thinned at high pressure with pressure-induced amorphization.

Remarkably, the XRD data of the decompressed sample showed obvious diffraction peaks, indicating that the recovered sample was crystalline, unlike its initial liquid state. The state of organic compounds is closely related to the number of C atoms they contain, for example, alkane will not be solid under ambient conditions if the molecule contains less than 16 C atoms. This suggests that the decompressed sample could be polymerized *p*-xylene since monomeric *p*-xylene only contains 8 atoms. Therefore, polymerization is likely to be the driving force of the phase transition around 13.5 GPa.

**Figure 4:**
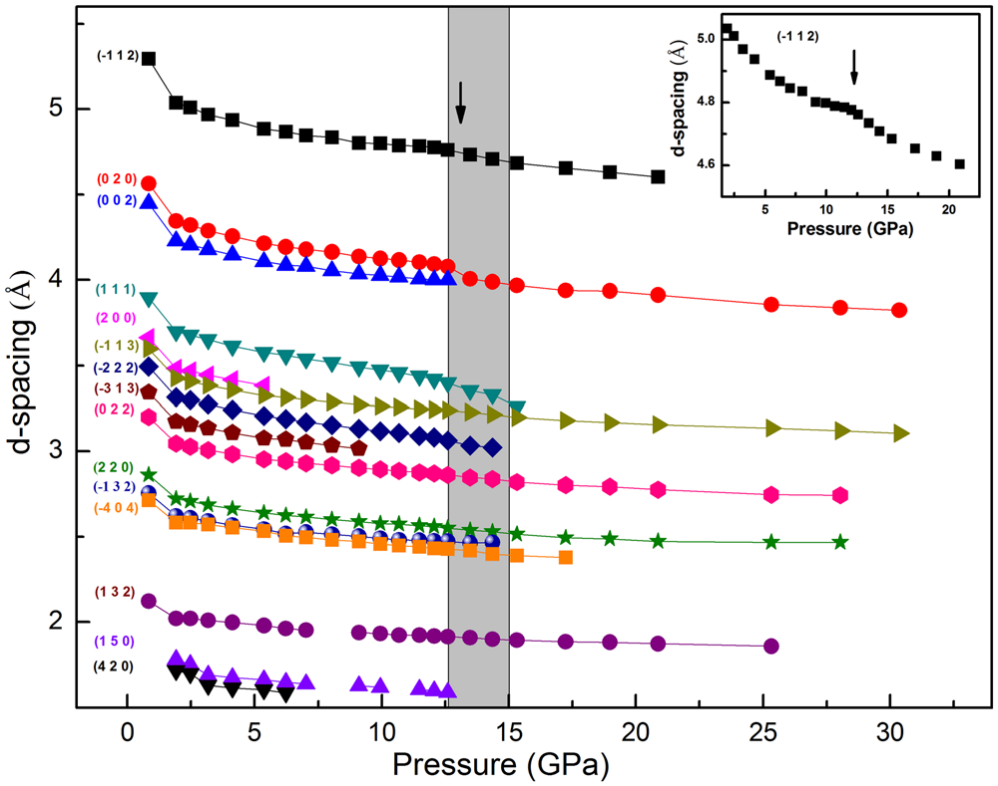
Pressure-dependence of the *d*-spacings from 0.8–30.7 GPa.

The pressure dependence of the *d*-spacings from 0.8–30.7 GPa is summarized in Fig. 4. It is clear that several *d*-spacings shrank sharply at around 13.5 GPa, which is consistent with the phase transition pressure, giving an insight into the transformation at this pressure. The molecules moved closer and closer as the pressure increased. The neighboring molecules started to transform as the pressure reached a critical value, and caused a much sharper decrease for some of the *d*-spacings, which may be due to polymerization in certain lattice directions.

**Figure 5:**
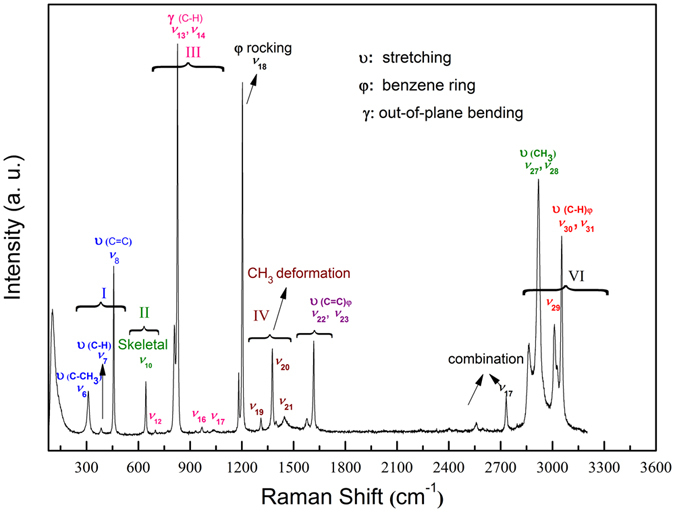
Raman spectrum of *p*-xylene from 80–3200 cm^−1^ under ambient conditions. The vibrational modes and assignments are also shown by groups.

Raman spectroscopy is a useful tool for examining the structures of materials^12–17^. While there have been studies of *p*-xylene with Raman spectroscopy under ambient conditions^18, 19^, investigations at high pressure have not yet been reported. Here, we performed Raman spectroscopy of *p*-xylene (80–3200 cm^−1^) up to 30.9 GPa at room temperature. Figure 5 shows the Raman spectra and major vibrational modes of *p*-xylene under ambient conditions. The Raman peaks for *p*-xylene are listed in Table 1 ^20^ and the Raman spectra are grouped based on these assignments, as shown in Fig. 5. Group I is assigned to the ring deformation modes, and three peaks were observed in this region. The skeletal vibration centered at 644 cm^−1^ is related to Group II. In substituted benzene ring compounds, the C−H out-of-plane bending vibrations^21^ give rise to bands in the region of 700−1000 cm^−1^. Four bands (Group III) were detected in this region and assigned to the C−H out-of-plane bending of the benzene ring. Group IV is assigned to the symmetry and anti-symmetry deformation of CH_3_, and Group V are those modes stretching the C = C of the benzene ring. The bands in the region of 2800−3200 cm^−1^ are group VI and assigned to the CH_3_ and C−H stretching vibrational modes.

**Table 1:** The Raman and infrared spectrum and assignments for *p*-xylene under ambient conditions.

Mode	Raman, cm^−1^	Intensity	IR, cm^−1^	Intensity	Assignments and approximate description
ν_6_	312	w			Ring deformation
ν_7_	386	vw			Ring deformation
ν_8_	458	s			Ring deformation
ν_10_	644	m			Skeletal
			795	vs	Skeletal
			1024	w	CH_3_ stretching
			1042	w	CH_3_ stretching
			1104	w	CH_3_ stretching
			1120	w	CH_3_ stretching
ν_12_	699	vw			C−H out-of-plane bending
ν_13_	810	m			C−H out-of-plane bending
ν_14_	827	vs			C−H out-of-plane bending
ν_16_	930	vw			C−H out-of-plane bending
ν_18_	1204	vs			Ring rocking
ν_19_	1312	w			CH_3_ asym. deformation
ν_20_	1377	m	1378	w	CH_3_ sym. deformation
ν_21_	1449	w	1454	m	CH_3_ asym. deformation
ν_22_	1577	vw	1516	s	C=C sym. stretching
ν_23_	1617	m	1631	vs	C=C asym. stretching
ν_25_	2559	vw			Combination/overtone
ν_26_	2732	w			Combination/overtone
ν_27_	2863	w	2869	s	CH_3_ sym. stretching
ν_28_	2919	s			CH_3_ asym. stretching
			2922	vs	CH_3_ sym. stretching
			2946	w	CH_3_ asym. stretching
			2975		CH_3_ asym. stretching
ν_29_	3011	m	3000	s	Benzene C−H stretching
ν_30_	3028	w	3020	s	Benzene C−H stretching
ν_31_	3053	s	3045	m	Benzene C−H stretching

**vs**: very strong; **s**: strong; **m**: medium; **w**: weak; **vw**: very weak.

**Figure 6:**
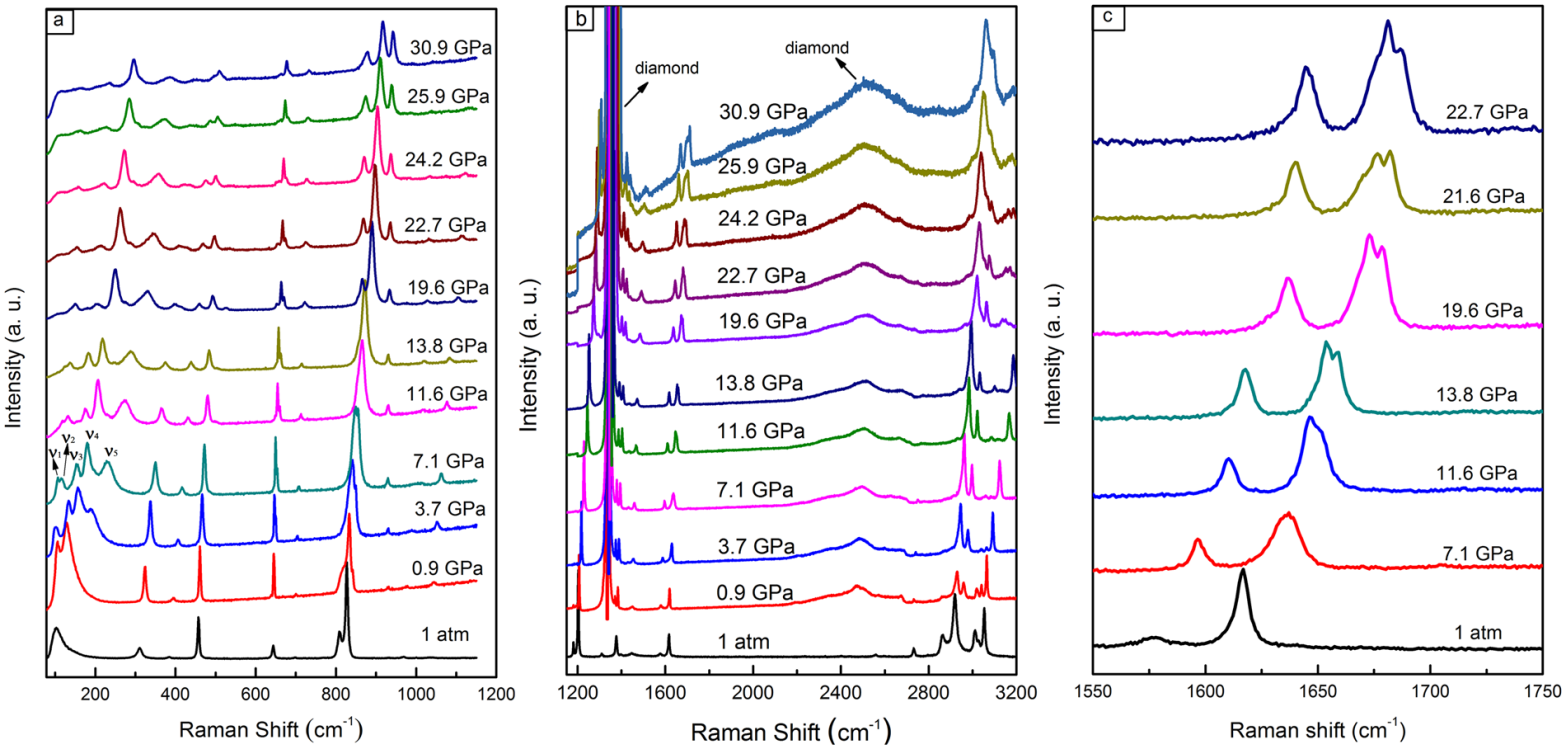
Raman spectra of *p*-xylene measured at various pressures and room temperatures. Spectra: (**a**) 80–1200 cm^−1^; (**b**) 1200–3200 cm^−1^; and (**c**) enlargement of 1550–1750 cm^−1^ at selected pressures. The strong bands centered at 1332 cm^−1^ and 2500 cm^−1^ are from the diamond.

**Figure 7:**
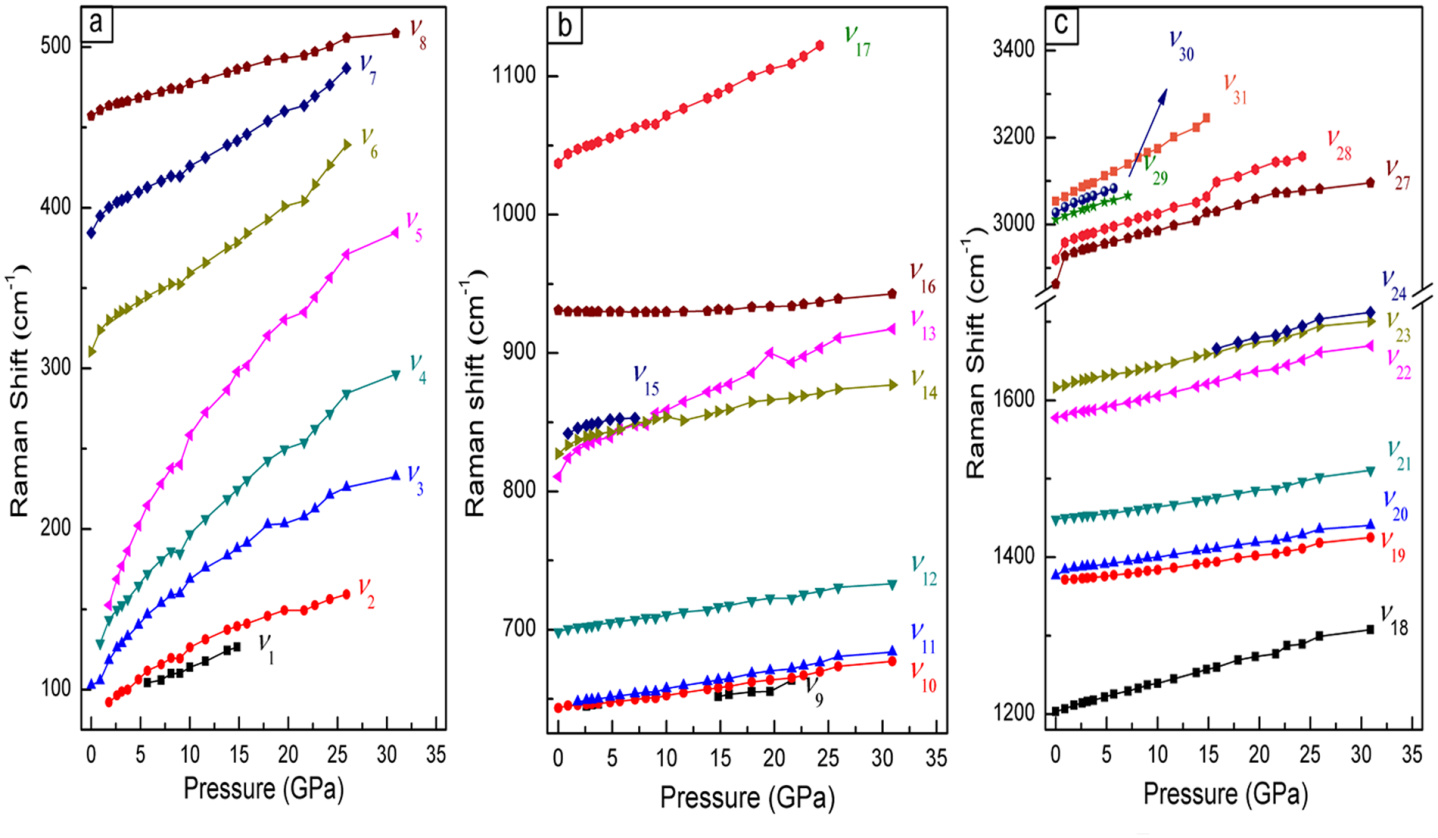
Pressure-dependence of the vibrational modes. Regions: (**a**) 75–525 cm^−1^; (**b**) 625–1150 cm^−1^; and (**c**) 1195–3500 cm^−1^.

Figure 6(a–c) presents the Raman spectra of *p*-xylene at various pressures. To better describe the pressure shift of the observed vibrational modes, the pressure dependence of the bands is summarized in Fig. 7(a–c). At 0.9 GPa, a new band appeared at 129 cm^−1^ (ν_4_) and was assigned to the lattice mode. This confirms the crystallization of *p*-xylene at high pressure. These changes are consistent with the crystallization observed by microscopy [see Fig. 1].

**Figure 8:**
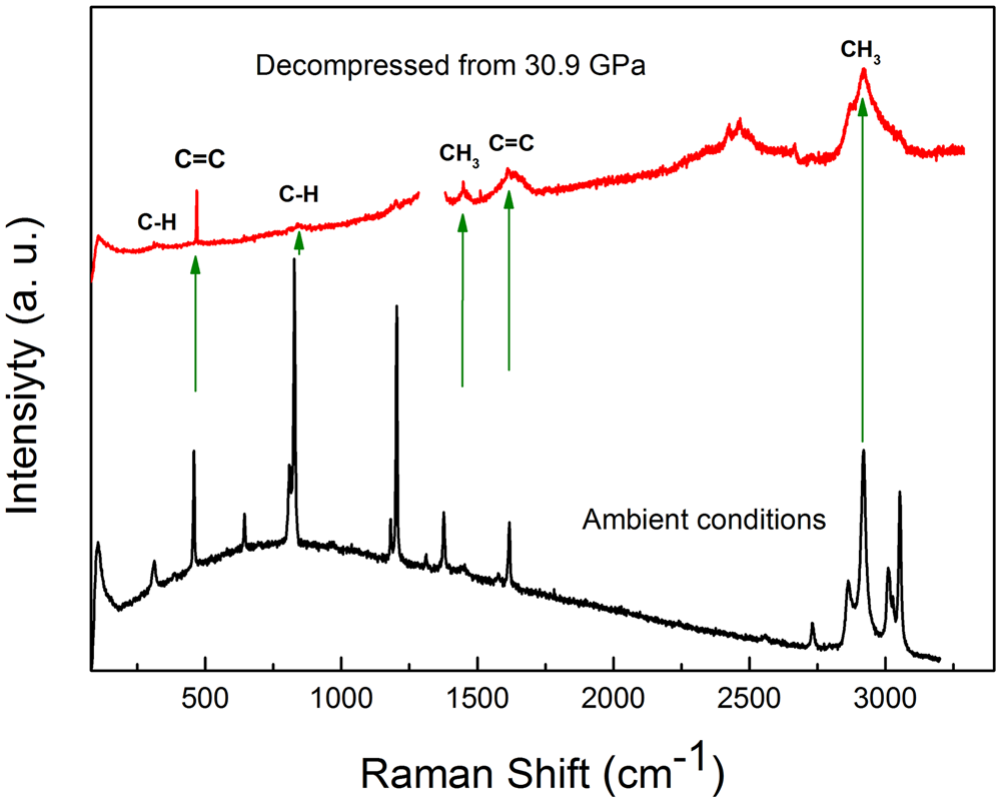
The Raman spectrum of the *p*-xylene at ambient conditions and decompressed from 30.9 GPa.

When the Raman spectra exceeded 13.8 GPa, the intensity of the C−H, CH_3_, and C=C stretching modes decreased, and the full width at half maximum (FWHM) increased. A band splitting appeared at 1657 cm^−1^, which was assigned to the anti-symmetry stretching of the benzene ring C=C. The enlarged view of the band splitting in the region of 1550–1750 cm^−1^ for the selected pressures is presented in Fig. 6(c). The polymerized phase was obtained from a number of triple- and double-bonded hydrocarbon compounds, such as acetylene and ethylene, at relatively low pressures (a few GPa). When the C=C bonds are involved in a ring, the system is more stable and expected to polymerize above 10 GPa. Therefore, the Raman changes above 13.8 GPa could be related to a new crystalline polymerized phase, as verified by the mass spectrometry investigation on the decompressed samples. These Raman changes were also consistent with the lattice shrinkage observed in the XRD. Furthermore, the ν_1_ band disappeared, bands ν_2_, ν_3_, ν_4_, and ν_5_ became well separated, and the intensity of bands ν_2_ and ν_3_, ν_5_ became very weak and broad [see Fig. 6(a)]. The peak weakening and broadening in the spectra suggested the presence of *p*-xylene strain, confirming the pressure-induced phase transition identified by XRD. The decompressed spectrum is clearly different from that at 1 atm as shown in Fig. 8, confirming that the transition is irreversible.

**Figure 9:**
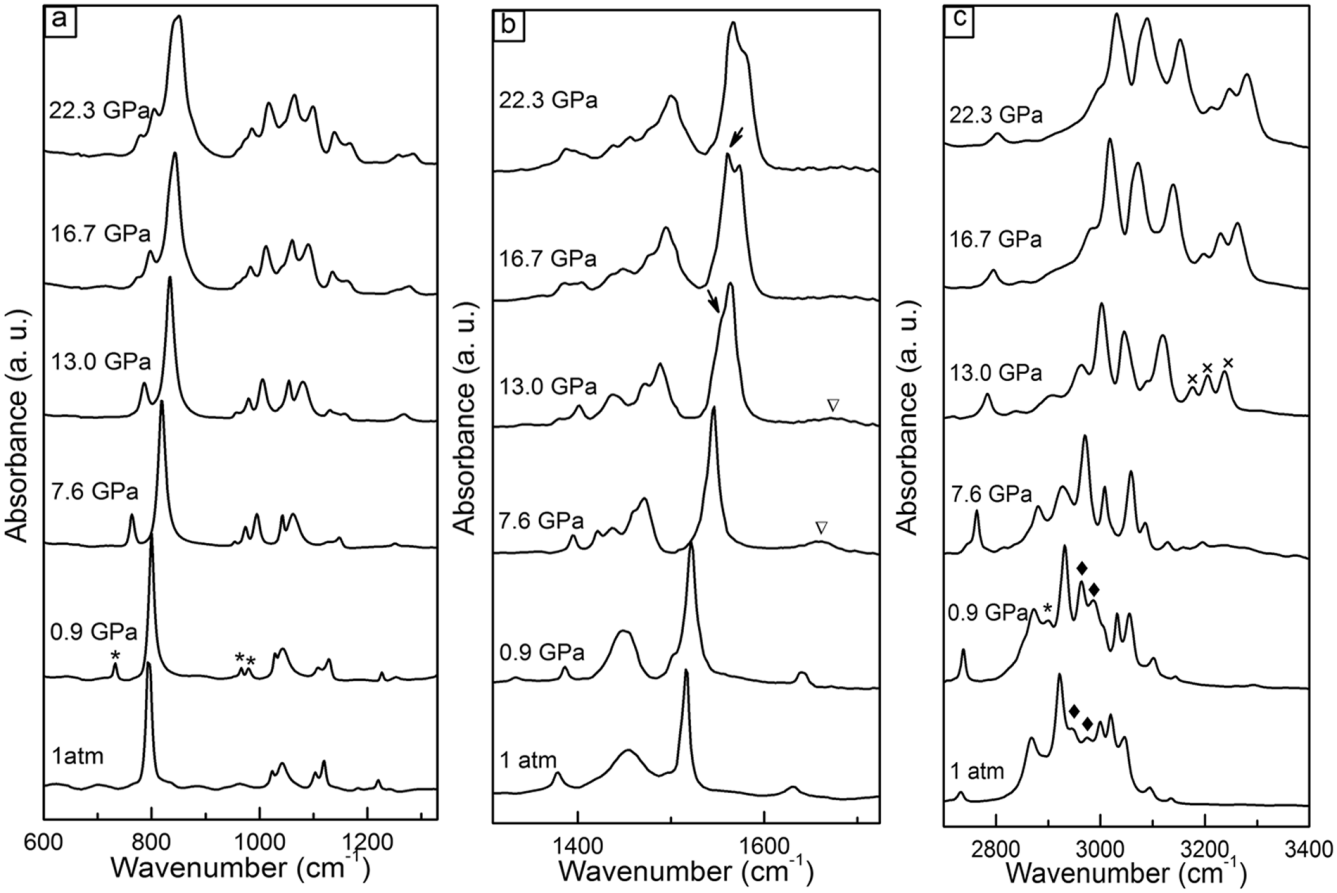
Evolution of the IR spectra of *p*-xylene measured under high pressure and room temperature: (**a**) Evolution of the skeletal and CH_3_ stretching region; (**b**) Evolution of the CH_3_ deformation and C=C stretching region; and (**c**) Evolution of the CH_3_ and C–H stretching region.

Infrared spectroscopy is also a powerful tool for characterizing polymers^22, 23^. We performed *in situ* high-pressure IR to investigate the pressure-induced crystallization and transformation processes, as shown in Fig. 9. Based on the assignments of the IR spectra in Table 1 ^24^, they were divided into three regions: (i) The skeletal and CH_3_ stretching vibration; (ii) The CH_3_ deformation and C=C stretching vibration; and (iii) The CH_3_ and C−H stretching vibration. At 0.9 GPa, four new peaks were observed, marked by asterisks. The presence of new peaks could arise from the enhancement of the intermolecular interactions due to crystallization^25^, which was confirmed by a micrographic observation and XRD^26^. The peak intensity increases marked by rhombuses in Fig. 9(c) were also due to the crystallization of *p*-xylene^27^. As pressure increased, obvious intensity changes were found, especially for the CH_3_ and C−H stretching vibration, as shown in Fig. 9(c). At 13.0 GPa, the CH_3_ stretching, CH_3_ deformation, and C=C stretching bands broadened, and a peak splitting appeared (marked by arrows) at 1555 cm^−1^. This splitting became much clearer at 16.7 GPa. To our knowledge, the splitting of the vibrational mode and the softening concomitant mode of the intramolecular modes are usually attributed to the formation of new intermolecular bonds^28–30^. Remarkably, the C=C stretching band at 1670 cm^−1^ disappeared and three new apparent peaks (marked by crosses) were observed at around 3210 cm^−1^, which was also due to new intermolecular bonds forming. Furthermore, we collected the IR spectra of the sample recovered from 33 GPa and compared it with the spectra at 1 atm. Note that the spectra at 1 atm and the spectra recovered from 33 GPa are different. Thus, the IR results confirm the pressure-induced transformation. The process of transformation involves the formation of new intermolecular bands, which relate to the C=C and C−H vibration of the benzene ring, and CH_3_ (deformation and stretching) vibration changes. This result is consistent with our Raman and XRD findings.

**Figure 10:**
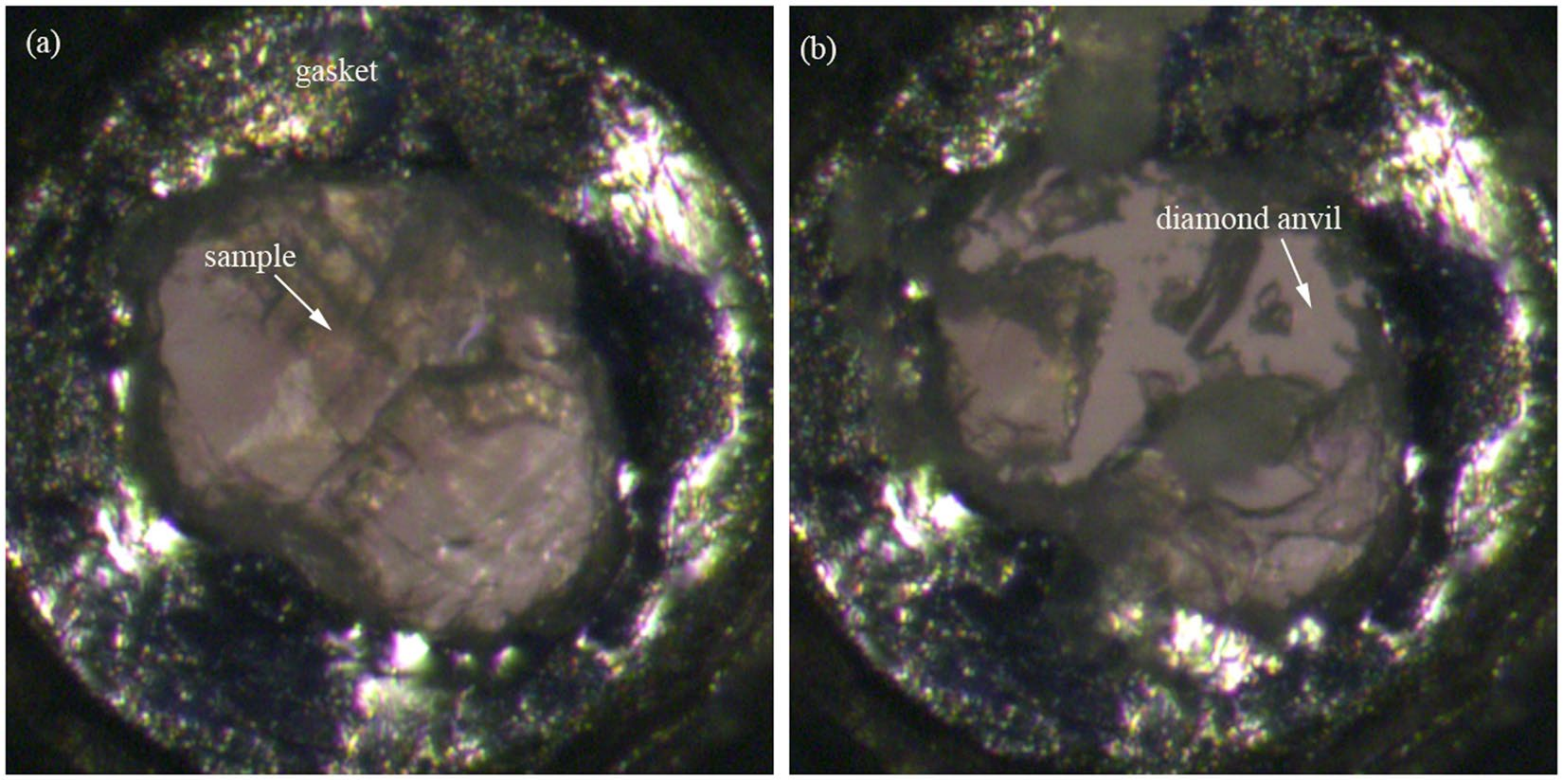
The final product of the *p*-xylene sample recovered from 20.1 GPa.

Next, we performed several additional independent experiments to confirm the new pressure-induced crystalline phase transformation. In these experiments, three recovered samples were obtained by recovering *p*-xylene from 12.6, 15.9, and 20.1 GPa at room temperature, respectively. We then compared the samples to each other. We found that the sample released from 12.6 GPa was liquid and finally evaporated when decompressed. However, the products recovered from 15.9 and 20.1 GPa were a transparent solid and crushed when the sample was poked with a needle, as shown in Fig. 10. The new phase could be sustained for a few months without further changes.

**Figure 11:**
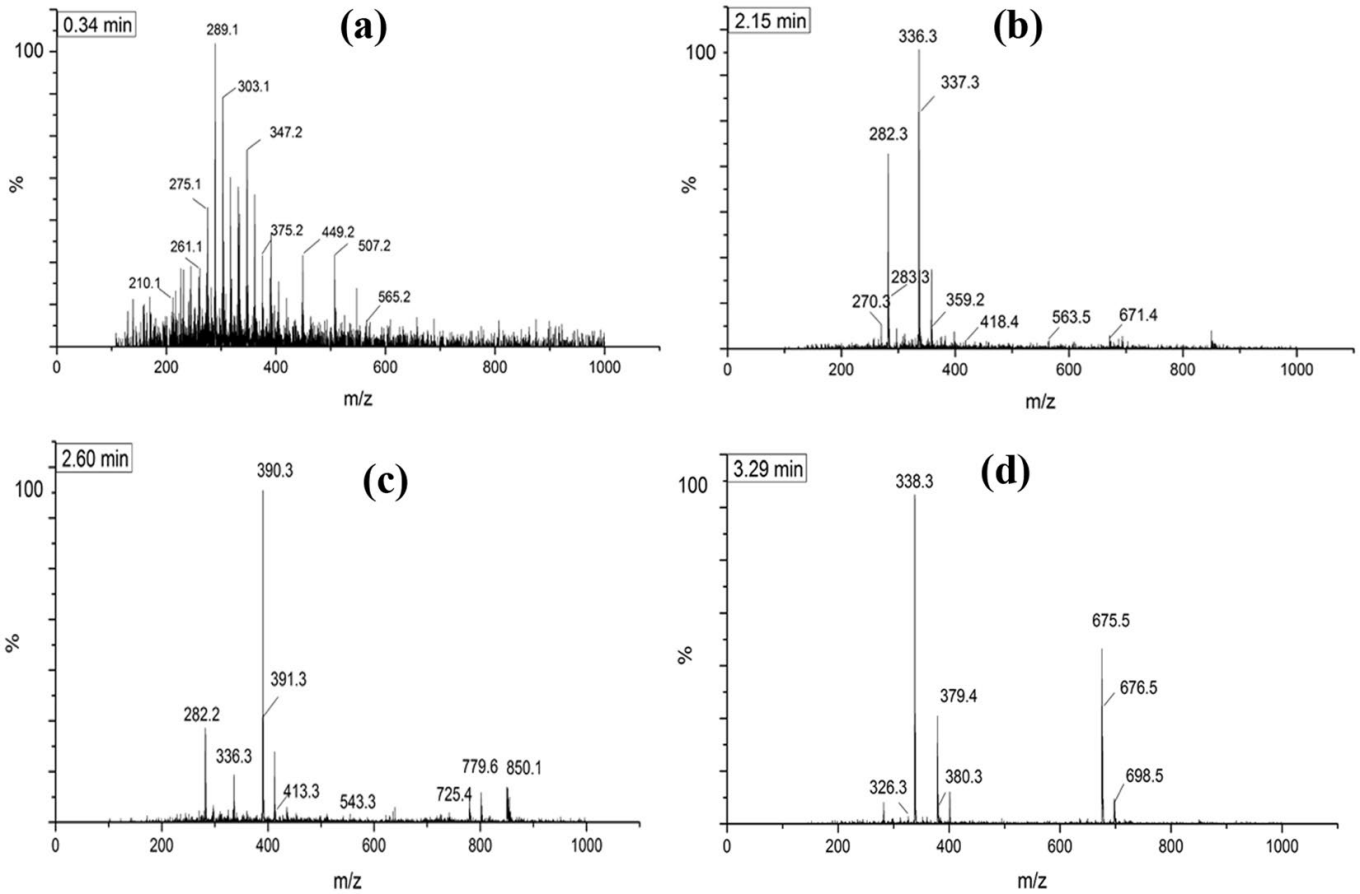
The mass spectra of *p*-xylene decompressed from 25 GPa to ambient conditions.

We also performed high-performance liquid chromatography (HPLC) mass spectrometry to investigate the decompressed *p*-xylene. The experiments were performed with a positive ion and the samples were decompressed from 25 GPa to ambient conditions. From the mass spectrum, a number of mass-to-charge (m/z, m is the mass and z is charge) appeared at m/z 289, 336, 390, 338, as shown in Fig. 11. Through analysis, we found that the m/z value of an oligomer is not exactly the integral multiple of the mass number of the C_8_H_10_ monomer. The random copolymerization and isomeric adjustment make the m/z of the polymer different. However, the oligomers mainly involved trimer and tetramer polymers. Therefore, we conclude that the application of pressure up to around 13 GPa does indeed lead to a new crystalline polymerized phase transformation of *p*-xylene.

## Conclusions

In conclusion, the crystallization and properties of *p*-xylene under high pressure were investigated by optical microscopy, XRD, Raman, and IR spectroscopy. *P*-xylene crystallized at ∼0.1 GPa with a monoclinic structure. Characteristic crystalline bands were identified by analyzing the Raman and IR spectra at 0.9 GPa. Our preliminary high-pressure Raman and IR analysis indicated that the pressure-induced polymerization occurred around 13 GPa. The new crystalline polymerized phase structure of *p*-xylene was characterized using results from XRD investigations at 17.2 GPa. Remarkably, the polymerized solid phase was quenchable to ambient conditions and confirmed by mass spectrometry experiments on the decompressed samples.

## Methods

High-pressure experiments were performed with a DAC equipped with a 300 μm culet. The gaskets were pre-indented to 40–50 μm with a 100 μm hole to serve as the sample chamber. The *p*-xylene sample (99.999% purity from Alfa Aesar Puratonic) was loaded into the DAC at room temperature (24–28 °C in our lab). A ruby ball was loaded with the sample to perform *in situ* pressure measurements via the R1 ruby fluorescence band shift^31^. The crystal growth and the new phase of *p*-xylene were studied with optical microscopy. Raman and IR absorption measurements were carried out using a 532 nm excitation laser and a Bruker Vertex80V FTIR spectrometer, respectively. The XRD experiments under high pressure and room temperature were performed at the BL15U1 beamline at the Shanghai Synchrotron Radiation Facility (SSRF) with a wavelength of 0.6199 Å and a CCD detector. The diffraction patterns were analyzed and integrated using the FIT2D program^32, 33^ to obtain the 1D intensity distribution as a function of the 2θ scattering angle. The mass spectrometry (MS) experiment was performed by high-performance liquid chromatography (HPLC) using an atmospheric pressure chemical ionization (APCI) source.
