# Author Correction: Pressure-Induced Crystallization and Phase Transformation of *Para*-xylene

**DOI:** 10.1038/s41598-018-26714-9

**Published:** 2018-06-14

**Authors:** Yanzhi Bai, Zhenhai Yu, Ran Liu, Nana Li, Shuai Yan, Ke Yang, Bingbing Liu, Dongqing Wei, Lin Wang

**Affiliations:** 1 Department of Physics and Astronomy and College of Life Science and Biotechnology, Shanghai Jiaotong University, Shanghai, 200240 People’s Republic of China; 2 Center for High Pressure Science and Technology Advanced Research, Shanghai, 201203 People’s Republic of China; 3 State Key Laboratory of Superhard Materials, Jilin University, Changchun, 130012 People’s Republic of China; 4 Shanghai Institute of Applied Physics, Chinese Academy of Sciences, Shanghai, 201203 People’s Republic of China; 5 High Pressure Synergetic Consortium, Geophysical Laboratory, Carnegie Institution of Washington, Argonne, Illinois, 60439 United States of America

Correction to: *Scientific Reports* 10.1038/s41598-017-05639-9, published online 13 July 2017

The Acknowledgements section in this Article contains errors.

“Portions of this work were performed at the BL15U1 beamline, Shanghai Synchrotron Radiation Source in China. The authors would like to thank the Shanghai Synchrotron Radiation Source for use of their synchrotron radiation facilities. This work was partially supported by the Natural Science Foundation of China (Grant No. U1530402, 11004072 and 11174201). The work was also supported by the Program for New Century Excellent Talents in University (NCET-10-0444), Ph.D. Programs Foundation of the Ministry of Education of China (20120073110057), and the State Key Laboratory of Explosion Science and Technology, Beijing Institute of Technology (KFJJ15).”

should read:

“Portions of this work were performed at the BL15U1 beamline, Shanghai Synchrotron Radiation Source in China. The authors would like to thank the Shanghai Synchrotron Radiation Source for use of their synchrotron radiation facilities. This work was partially supported by the Natural Science Foundation of China (Grant No. U1530402, 11004072 and 11174201). The work was also supported by the Program for New Century Excellent Talents in University (NCET-10-0444). Dong-Qing Wei is partially supported by the Science Challenge Program No. TZ2016001 and the State Key Laboratory of Explosion Science and Technology, Beijing Institute of Technology (KFJJ15).”

